# LAI-ART Awareness, Willingness, Barriers and Facilitators among Black Sexual Minority Men Living with HIV in the US South

**DOI:** 10.3390/ijerph21050602

**Published:** 2024-05-08

**Authors:** Chadwick K. Campbell, Kirstin Kielhold, Hannah E. Reynolds, Wilson Vincent, Daniel E. Siconolfi, Stephen D. Ramos, Adedotun Ogunbajo, Susan M. Kegeles, Erik D. Storholm

**Affiliations:** 1 Herbert Wertheim School of Public Health and Human Longevity Science, University of California San Diego, La Jolla, CA 92093, USA; kkielhold@ucsd.edu (K.K.); sramos5@sdsu.edu (S.D.R.); 2 School of Public Health, San Diego State University, San Diego, CA 92182, USA; hreynolds7166@sdsu.edu (H.E.R.); estorholm@sdsu.edu (E.D.S.); 3 Department of Psychology and Neuroscience, Temple University, Philadelphia, PA 19122, USA; wilson.vincent@temple.edu; 4 RAND Corporation, Pittsburgh, PA 15213, USA; dsiconol@rand.org (D.E.S.); ogunbajo@rand.org (A.O.); 5 Department of Medicine, University of California San Francisco, San Francisco, CA 94158, USA; susan.kegeles@ucsf.edu

**Keywords:** long-acting injectable ART, acceptability, willingness, black sexual minority men, HIV treatment, adherence

## Abstract

Black sexual minority men (BSMM) continue to bear a disproportionate burden of HIV in the United States, with the highest incidence and prevalence in the southern region of the country. In Texas, BSMM living with HIV (BSMM+) have the lowest rates of viral suppression of all SMM and have lower antiretroviral treatment (ART) adherence than white and Hispanic SMM. Long-acting injectable ART (LAI-ART) can potentially overcome several barriers to daily oral ART adherence (e.g., stigma, forgetfulness, pill fatigue). However, little is known about the knowledge, willingness, barriers, and facilitators regarding LAI-ART among BSMM+. From July 2022 to September 2023, we conducted in-depth, semi-structured interviews with 27 BSMM+ from the Houston and Dallas Metropolitan Areas, Texas. Data were analyzed using a thematic analysis approach. Most men knew about LAI-ART, but their understanding varied based on their existing sources of information. Some men were enthusiastic, some were cautious, and some reported no interest in LAI-ART. Barriers to LAI-ART included a lack of public insurance coverage of LAI-ART; fear of needles and side effects; the frequency of injection visits; the requirement of viral suppression before switching from oral ART to LAI-ART; and satisfaction with oral daily ART. Motivators of LAI-ART uptake included the eliminated burden of daily pills and reduced anxiety about possibly missing doses. BSMM+ may be among those who could most benefit from LAI-ART, though more research is needed to understand which factors influence their willingness and how the barriers to LAI-ART might be addressed, particularly among diverse communities of SMM of color.

## 1. Introduction

Black sexual minority men (BSMM) (e.g., gay, bisexual, queer) continue to bear a disproportionate burden of HIV in the United States (US), with the highest incidence and prevalence in the southern region of the country [1]. In the Southern region of the US, generally, only 52% of people living with HIV (PLWH) were retained in care and only 63% achieved viral suppression in 2020 [2], and, indeed, BSMM living with HIV (BSMM+) in the South have less than optimal antiretroviral treatment (ART) adherence and viral suppression [2,3]. In Texas, BSMM+ are significantly less likely to be adherent to ART compared to white and Hispanic SMM and less likely to have achieved sustained HIV viral load suppression than white SMM [4].

Several individual, social, and structural barriers to oral ART adherence exist among BSMM+. Studies have documented BSMM+ identifying pill forgetfulness and changes to normal routines as major facilitators of missed medication doses [5,6]. For others, taking a daily pill can be a constant reminder that they are living with HIV [7], which can interfere with their experience of taking ART. Experiences of trauma, including childhood sexual abuse, intimate partner violence (IPV), and other forms of violence, have a negative influence on ART adherence and HIV treatment outcomes among SMM [8,9,10,11]. The sequalae of traumatic experiences can include poor mental health or substance use, both of which are associated with poorer adherence and viral non-suppression [9]. In addition, HIV stigma is known to contribute to poor adherence and a lower likelihood of viral suppression [9,12,13]. HIV stigma has led some to skip ART doses due to the concern that someone who is unaware of their status may see their pill bottle, thereby inadvertently revealing their status [13,14,15]. Together, these documented barriers to optimal ART adherence point to the utility of alternative dosing strategies and formulations that can enhance the adherence to ART among BSMM+.

Long-acting injectable ART (LAI-ART) has the potential to overcome some of the known barriers to oral ART adherence and sustained viral suppression [16]. Specifically, LAI-ART can eliminate the burden of remembering to take daily oral doses, particularly at times of acute stress or when travelling [17]. Furthermore, LAI-ART could eliminate concerns about hiding pill bottles from those who are unaware of one’s seropositive status, and daily oral ART would no longer serve as a constant reminder of one’s HIV status [7,18,19,20]. To date, most research on long-acting formulations among SMM has focused on LAI pre-exposure prophylaxis (LAI-PrEP) [21,22,23,24,25]. However, little research has examined the knowledge and awareness, or the barriers and motivators, regarding LAI-ART uptake among BSMM+. Further, the research that has been conducted on this topic was overwhelmingly conducted prior to the FDA approval of LAI-ART [16,17,18,26]. To our knowledge, no such research has been conducted in the US South, and there is a dearth of research exploring these topics since they have become available as an FDA-approved treatment option. With the current study, we began to address this gap. We sought to examine the knowledge and awareness of and willingness to use LAI-ART, as well as the motivators of and barriers to LAI-ART uptake among BSMM+ in the US South.

## 2. Methods

Between July 2022 and September 2023, the first author and qualitative study PI (CKC), who self-identified as a Black gay man from the US South, conducted in-depth, semi-structured interviews with 27 BSMM+ in the Houston and Dallas Metropolitan Areas, Texas. Participants were recruited from a community-based, longitudinal cohort originally recruited between September 2015 and November 2016, primarily using long-chain peer referral [27]. Details of the longitudinal cohort study procedures have been previously reported [28,29]. At the time of recruitment, eligible participants were 18–29 years old; identified as Black/African American; reported sex with a man in the last year; and were living with HIV. HIV status was confirmed using a rapid oral test for HIV (INSTI Rapid HIV-1/HIV-2 Antibody Test; bioLytical Laboratories, Inc., Richmond, British Columbia, Canada). Quantitative, longitudinal data were collected at four waves: September 2015–November 2016; January–September 2018; October 2019–March 2020; and May 2022–October 2022.

Participants in the qualitative interviews were purposively selected from the fourth wave of data collection to include men who reported suboptimal care engagement (e.g., reported missing ART doses in the past 12 months, detectable or unknown viral loads, or not currently taking ART) or who had experienced IPV or substance use. We stopped collecting data after 27 interviews once we determined that we had “information power”, a process in which the researcher makes a judgement that the data are sufficient for the proposed analysis [30], which is considered more appropriate for thematic analysis than the concept of saturation [31]. Interview domains included HIV stigma (e.g., What’s it like to be a Black gay man living with HIV in your community? What kinds of things do people say about HIV and people living with HIV?); HIV diagnosis and experience living with HIV (e.g., Please walk me through the experience of being diagnosed with HIV, How has your life changed since you were diagnosed); factors influencing HIV care engagement (e.g., What kinds of things make it more difficult to take your medications? What, if anything, do you think would improve your ability to stay engaged in your HIV healthcare?); intimate partner violence (e.g., Have you noticed an increase in anger or frustration with each other because of issues related to COVID? How has relationship violence or abuse affected how and when you engaged in HIV care?); and the effects of COVID-19 on care engagement and other general impacts on their lives (e.g., Tell me about how the pandemic has affected your relationship with your HIV doctor/provider? Generally speaking, what has the pandemic been like for you?).

The Institutional Review Board at San Diego State University reviewed and approved all study procedures. All participants were sent a copy of the consent form prior to the scheduled interview. The interviewer reviewed the consent form and answered participant questions, and each participant provided verbal consent prior to beginning the interview. To protect participant privacy, we obtained verbal consent, such that no qualitative study data were directly associated with a participant’s identifying information. Data were only available to members of the study team, and we have used pseudonyms in the reporting of qualitative findings. Participants received a USD 75 gift card for their time and contribution to the study.

The first author conducted all interviews, which lasted 60–90 min, on average, and were audio-recorded and transcribed verbatim. All interviews were conducted virtually over Zoom. Three analysts (CKC, HER, and KK) conducted the qualitative analysis using a codebook thematic analysis (TA) approach, which, within the spectrum of TA, is located between the postpositivist and constructivist paradigms [32]. Thematic analysis is a flexible qualitative approach that can elicit complex, rich, and detailed data [33], and a codebook thematic analysis approach is appropriate when the research questions that one is aiming to answer are very clear, as they were in our study [32]. First, the three analysts listened to the interviews and audited the transcripts to become deeply familiar with the data. Each analyst took notes and identified potential codes during this process and discussed them during weekly meetings. The three analysts then iteratively, independently conducted open coding on three transcripts. The analysts met after each transcript was coded to compare and discuss the codes, while consolidating similar codes and identifying parent codes to categorize child codes into a codebook. Next, the three analysts independently coded the transcripts, while discussing potential themes and, in line with coding thematic analysis, making necessary modifications to the codebook throughout the analysis process [34,35]. Decision trails documenting codebook development, team discussions, and codebook revisions were maintained throughout the process.

## 3. Results

Most men in our qualitative sample identified as gay (78%), with 22% identifying as bisexual, and were between the ages of 26 and 35 (median = 31) years old at the time of the interviews. At the time of the quantitative survey, the majority had at least some college education or an associate degree (52%), were employed (59%), earned less than USD 40,000 in annual income (59%), and had health insurance (70%). Participants had been living with HIV for an average of 10.85 years. While a large majority (85%) reported currently taking ART, more than a third (37%) of these reported missing an ART dose 2–3 days a week or more in the previous 60 days, and 23% reported a detectable or unknown viral load. More than half (56%) reported using one or more substance in the past 60 days, and, of the 22 men who reported having ever been in a relationship, 77% (*n* = 17) reported having been in at least one relationship where violence or abuse occurred. All participant demographics are shown in Table 1.

**Figure 1:**
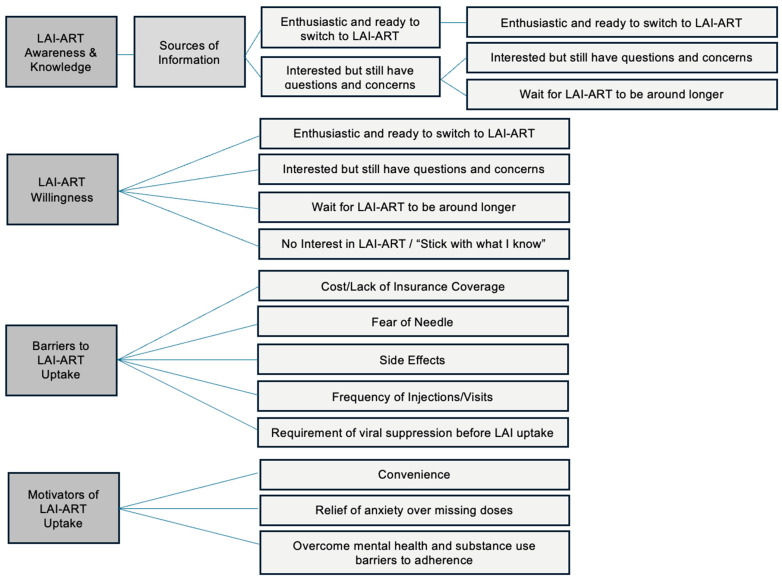
Thematic analysis map of findings.

In the results that follow, we present three broad themes: (1) LAI-ART awareness and willingness; (2) LAI-ART willingness; (3) barriers to LAI-ART uptake, which includes three subthemes—cost and insurance coverage, needles and side effects, and frequency of visits; and (4) motivators of LAI-ART uptake. The results of the thematic analysis are illustrated in Figure 1. All names associated with quotes are pseudonyms, and quotes are followed by (participant pseudonym; age; viral load at fourth wave of survey).

### 3.1. LAI-ART Awareness and Knowledge

Nearly all (26/27) participants reported being aware of LAI-ART, though with varying levels of knowledge. One participant had no awareness of LAIs, and one was on LAI-ART at the time of the interview. Some reported only being aware of them from pharmaceutical commercials, and, consequently, these men tended to report having only limited knowledge: “I’ve heard of some, but I never actually look into it… I just saw the commercials and things on the posters” (Stephen; 31; Undetectable). Importantly, men described that the limited information in commercials did not convince them that they should try LAI-ART. As Kevin described, “I haven’t heard anything past the commercials because my doctor hasn’t brought it up at all and nobody at [community health care center] has brought it up at all. I’ve only heard stuff through the commercials and a commercial ain’t swaying me”. Commercials served to introduce LAIs but did not provide information about how they work or any associated risks: “I’ve seen it on TVs and YouTube commercials. As far as me like diving into studying it? No, I haven’t. I haven’t really studied it, but I mean, if that’s an option, I can look into it, but I don’t want to be a guinea pig. I really just heard about the side effects. Like I said, I just seen it on a commercial or on YouTube but it’s an ad, so I always hit skip” (Keith; 30; Undetectable).

In contrast, some learned about injectable treatments from friends who were currently on LAIs. Jesse had heard about them from his doctor but felt that it was good to have a friend he could ask directly.

Recently, me and my friends, we’ve been talking about [HIV] because they’re getting this new shot that they have to take every month or every other month or something… You know, I heard about it from my doctor but knowing somebody who actually is doing the shot…

(Jesse; 28; Detectable)

As Jesse suggests, men found value in learning about LAIs from trusted members of their social network. When asked what he had heard about LAIs, he described hearing “that it’s good, because I have a friend who does it” (George; 32; Undetectable). In contrast to men who had only heard about LAIs from commercials, friends were able to provide more information.

I heard of the shot. I think it’s in the butt... I have a friend that, he’s on it, and he’s a little bit older than me, but he takes the shot. He said at first it kinda hurt, it felt like a penicillin shot, but then he was like, when you start going, you get used to it. I think he said it’s like every eight weeks, and then it in both cheeks. He likes it, he said it’s better than taking medicine every day.

(Brandon; 29; Undetectable)

As these quotes suggest, the source of information could be an important factor in whether men have accurate knowledge about and trust in the safety of LAI-ART.

### 3.2. LAI-ART Willingness

Most participants expressed some willingness toward switching from daily oral ART to LAI-ART. About a third (*n* = 8) were enthusiastic about making the switch if and when they had the opportunity. Gerald described, “I wanted to talk to my doctor about it next time I see her because I think that would be great and easier to manage” (Gerald; 32; Undetectable). Others felt that the shot would be better than taking a pill if they were able to have access to LAIs.

If it’s covered with insurance and if I’m able to get it at no cost, honestly, then I would be, I’d be down to do it. Because I’m pretty open to trying new things, that does sound a lot better than be having to take a pill every single day, I would be interested.

(Jamar; 26; Undetectable)

Similarly, Edwin expressed that he planned to explore how he could access LAIs:

I have heard of this, and I have wanted to know how to get in access or contact with the person that knows about this, because it sounds like something I want to try, because if it’ll take me off of pills, I’m down.

(Edwin; 33; Undetectable)

While the largest group of participants (*n* = 12) were interested in exploring LAI-ART, they had unaddressed concerns that would significantly influence their decision to switch from daily oral ART to LAI-ART. For example, Anthony described some of the questions that he would want to have answered while considering a switch to LAI-ART.

I have questions because I want to know how is this once every two-month drug effective versus something that you’ve been taking every day or that you’ve had to take every day? Is it an extended release? Is it a higher dosage? Is it more potent, I guess? I don’t know. I’m like, okay, if it’s a stronger drug, because it has to be stronger if you’re going to be getting it once every two months.” So how is that going to affect you when you first get it? Are you going to have the downtime or what? So it’s questions that I have regarding that.

(Anthony; 30; Undetectable)

Some simply wanted to wait until LAIs had been publicly available longer, so that there was time for them to observe the potential benefits and risks among others.

That was something I wanted to talk to [my doctor] about. I just know I still want to give it maybe a couple more months, like eight months to a year, before I make a complete decision. I just want to see what goes on. They probably been testing this for a long time… I guess just to see how more people react to it. Versus just seeing what you see on TV and what you see on social media.

(Jason; 34; Undetectable)

Other participants wanted assurance that they would remain undetectable and would continue to be healthy if they made the switch. Importantly, some had misunderstandings about how LAI-ART works. For example, Kevin worried about stopping oral treatment and immediately beginning injections, as this would not leave time to assess the side effects and make adjustments to his medication as needed: “I know going from a pill to an injectable that’s much more directed in your bloodstream, that’s going to really, probably, have to be an adjustment to make, side effect wise…” (Kevin; 28; Undetectable). After being told, during the interview, that people switch to the new drug in pill form for a period of time first, he expressed, “I’m way more reassured hearing that you have to do a pill form first”.

Lastly, a small number of participants expressed no interest in switching to LAI-ART. They expressed satisfaction with their oral treatment and preferred to stick with what was already working for them. Some expressed reservations about injections, as Craig emphasized, “I stick to what I already got, what works for me. I don’t want to change my remedies or my witchery, or none of that. I just want the medicine that I know. I don’t want to start nothing new… No. I don’t want to inject nothing in me” (Craig; 30; Undetectable). Kevin simply felt more comfortable sticking with his current treatment.

I’ve been on Triumeq for so long and it’s worked well with me for so long, I would just be leery, not anti, but leery of an injectable... My pill works. It took me a long time to get used to my pill and now I’m used to it and I know it agrees with me now. And I don’t have to worry about trying something new and worrying about it if it agrees with me or if I have to even go back to my pill.

(Kevin; 28; Undetectable)

For the men not interested in using LAI-ART, they were not interested in taking a risk with a new treatment approach that could cause side effects or not work as well.

### 3.3. Barriers to LAI-ART Uptake

Cost/Insurance Coverage. Several participants who expressed interest in switching to LAI-ART noted that they had been told by their doctors that LAI-ART would not be covered under public insurance programs in Texas. Jesse described the conversation that he had with his doctor: “So, the doctors tell me the insurance really don’t want to pay. But I feel like, I mean, it’s still fairly new, so I feel like, after a while, the insurance probably will help more” (Jesse; 28; Detectable). Paul believed that he would never have access because of his insurance:

I inquired about it and my doctor told me I’ll never see it because she was like, “it costs so much that, there’s not going to be a program for it, and you’ll have to pay for it out of pocket, and it would just be expensive”, because I inquired about it… Yeah, because like we got Ryan White and then they got other stuff, then they got other programs that pay for the medicine, but she was like, when it comes down to that one, she was like, they don’t have a program for it.

(Paul; 26; Detectable)

Several others described being given the same information when they asked their providers about switching to LAI-ART. Marcus considered enrolling in a study so that he could access LAIs before his insurance approved it.

I’m trying to get it. My insurance doesn’t qualify for it, but I’m looking maybe for a study, or something. Something I want tomorrow if I could do it… So, they don’t approve the shot yet. So that’s all I’m waiting on.

(Marcus; 30; Undetectable)

Needles and Side Effects. A few participants expressed how their fear of needles would make them hesitant to switch to LAI-ART. As Mason described,

I’m not a fan of needles. I don’t like needles and stuff like that… I wonder how thick the needle is and the medication. I imagine there got to be a lot going inside of you at one time… If I heard a complication from a person, I’m not going to do it.

(Mason; 31; Undetectable)

Others worried about the potential side effects of a new medication, especially compared to their current daily oral ART regimen: “I think it’s just that, I like my Triumeq. I don’t want to have to take another the medicine. And then that be some type of side effect because that would be my luck” (Aaron: 26; Undetectable). However, most of these men also suggested that, with more time and information, they would still be willing to consider LAIs.

Frequency of Visits. A few participants noted that having to go to the doctor’s office every two months might be a difficult commitment. For example, Anthony worried that the frequency would interfere with his job: “I’m like once a month or once every two months… Ah, don’t want to commit to that. And then you have to take off to work to go get it done too” (Anthony; 30; Undetectable). Similarly, George felt that his inconsistent work schedule would make it difficult to attend more frequent medical appointments.

The reason why I haven’t started on it is because I don’t… The way my work schedule was, you only got so many days in between… So, I didn’t want to take that risk. So, once I can kind of get my work schedule down pat, then, yeah, I think that’s what I’ve been wanting to do.

(George; 32; Undetectable)

Requirement of Viral Suppression Before Initiating LAI-ART. Just under a quarter (23%; *n* = 6) of the men in this subsample reported having a detectable or unknown viral load at the time of the quantitative survey. At the time of the interview, half of these men had re-engaged in care, started taking oral ART again, and were undetectable. Two men who still had a detectable viral load when they were interviewed described that they were interested in LAI-ART but were unable to start LAIs because they had detectable viral loads (when the interviews were conducted, the LAI prescribing guidelines required that an individual had achieved viral suppression using oral ART before transitioning to LAI-ART). For example, Phillip described,

I know I’ve been looking into this, you know, injection medicine, you know, that they started coming out with where you can go and get a shot once a month. It looks good, but I’m trying to—now my focus is I’m trying to get the medicine in my system so that—because they told me the only way I’ll be able to switch to an injectable, if it’s even available at a clinic I go to, is I have to get to undetectable first. That’s a requirement—so, that’s my focus now.

(Phillip; 35; Detectable)

Jamal also described struggling with oral ART, but could not switch to LAIs because of his viral load.

I like living free and just going about life without having to be, I guess you could say, responsible and take a pill every damn day or what have you… I would much rather move to, like, the injection once every three months or once every six months, but you have to get to undetectable in order to do that. But, yeah, just the whole pill idea feels restrictive to me because you have to keep them…

(Jamal; 35; Detectable)

### 3.4. Motivators of LAI-ART Uptake

Participants who expressed an interest in LAI-ART were asked about their motivations to switch to an injectable treatment. Some emphasized the convenience of the injection and that it would eliminate the daily burden of taking a pill. Jesse explained that he “would love to go take a shot every other month versus a pill every day” (Jesse; 28; Detectable). Similarly, Stephen felt that injectables would be easier. “One shot? A little poke for at least 15 or 20 s is better than taking something down your mouth every day” (Stephen; 31; Undetectable). Others wanted relief from anxiety about missing doses. Marcus expressed wanting to know that he was always virally suppressed.

I just feel I would be undetectable. That’s all I want, want to make sure I wake up every day, and I’m undetectable. Because I know if I miss something, then I’d be worried… As long as I hear I’m undetectable that I’m happy, that’s all I want. That’s all I want to hear. And I think if I get the shot, I’ll be fully undetectable, I think.

(Marcus; 30; Undetectable)

Importantly, some of those who reported struggles with adherence due to depression or substance use acknowledged that LAI-ART would inherently reduce their missed doses during periods of heavy drug use because of the longer-term dosing window (bimonthly versus daily). For example, Keith described,

You know I wouldn’t mind doing it, due to, there are days that I do forget to take my medicine or there are days where I go out and I binge, and I don’t have my medicine with me. So, you know, that would be very beneficial. But I’m always like worried about the side effects or what’s going to happen to me.

(Keith; 30; Undetectable)

Similarly, Phillip, who had a history of substance use and ongoing medical issues that often left him tired and in pain, felt that injectables would help him to better manage his HIV when he was having a challenging day.

I feel like it would be easier for me to go to my doctor and get my shot, you know, once a month or whatever frequency they want me to. It’s easier that way for me. And then I wouldn’t have to remember every day. And then, some days, you know, when I’m sick it’s like I don’t even want to get up out the bed.

(Philip; 35; Detectable)

## 4. Discussion

Our qualitative findings can inform interventions aimed at increasing the uptake of LAI-ART among BSMM+ who can most benefit from this new technology, especially those with suboptimal adherence to daily oral ART. Much research has explored the knowledge, willingness, barriers, and facilitators regarding LAI-PrEP uptake among BSMM [23,36,37,38], but little is known about LAI-ART among BSMM+. All but one participant in our study were aware of LAI-ART, though the overall level of self-reported knowledge varied according to the source of their information. Importantly, those who learned about LAI-ART from members of their social network on LAI-ART were able to hear about their friends’ level of satisfaction with LAI-ART and believed they were more informed about how injectable treatments work. Those who had only learned of LAI-ART from advertisements were aware of them but were less informed about the details of the LAI process. Further, for some, peers are more convincing sources of information than public health authorities or pharmaceutical companies. Learning from peers who have experience with LAI-ART may be an effective way to ensure that those who may be interested in new HIV treatment options receive the information that they need to make decisions about treatment modalities.

For some men, oral daily ART was seen as a burden from which they wished to be relieved. Reasons included having to remember daily pills, feeling anxiety about possibly missing doses, and desiring the opportunity of “living free”. However, there are also several barriers that hinder the accessibility of these newer treatment formulations. In line with previous research [16,18,26,39], some men in our study expressed concerns about needles and the potential side effects of LAI-ART. Notably, the participants in our sample who expressed some caution about LAI-ART were not altogether opposed to the possibility of switching to LAIs. Instead, they expressed that they would be open to considering LAI-ART if they had more information. Some men wanted to wait until there had been greater public uptake, and others had some questions that they wanted to be answered first. Moreover, some had misunderstandings about the process that could be easily addressed, such as Kevin’s belief that long-acting injections begin immediately after discontinuing one’s current oral regimen. Some men expressed having no interest in switching to LAI-ART because they were satisfied with oral ART. Others worried about the newness of LAI technology or were simply opposed to changing to “witchery” and did not “want to inject nothing” into their bodies, which, to some extent, may reflect well-documented medical mistrust among Black Americans [17,26,40,41].

The participant narratives also highlighted a number of structural barriers that may be more difficult to overcome than individual awareness, knowledge, attitudes, or behaviors. First, men who struggled to reach and maintain viral suppression were aware that their detectable viral load prevented them from being able to initiate LAI-ART. While only a couple of men in our sample discussed this barrier explicitly, we found it important to include in our findings as these men represent some of the PLWH who could most benefit from LAI-ART. In both cases, these men experienced problematic substance use and felt that managing daily oral ART was difficult in the context of substance use. The current guidelines require that patients achieve viral suppression before starting LAI-ART formulations, as the clinical trials for LAI-ART only enrolled people who were virally suppressed [42,43,44]. This leaves those who could most benefit from LAI-ART unable to access this critical new treatment option. However, some data have shown that LAI-ART is just as effective in achieving viral suppression among those who are not virally suppressed as among those who are [45,46]. Second, at the time of these interviews, and as of the preparation of this article, the state of Texas did not cover LAI-ART as part of their HIV drug formularies [47,48]. Most men in our qualitative sample were on public health insurance at the time of their interviews. Indeed, several men had been told by their doctors that they would not be able to switch to LAI-ART unless they were able to pay out of pocket or obtained private insurance coverage. Lastly, some men worried that the frequency of visits to receive ART injections provided in clinical settings might be difficult to manage—a potential barrier to LAIs noted by others [49,50]—particularly when one has an inconsistent or unpredictable work schedule.

Though this work has many strengths, there are limitations to our study worth noting. First, we interviewed BSMM+ in two large metropolitan areas in the state of Texas. Each of these cities has large populations of Black persons, relative to the Black population of the US, and large LGBTQ populations; thus, generalizations to BSMM+ living in smaller cities and towns may be limited. Second, structural barriers to LAI-ART, such as public health insurance formularies, are shaped, in part, by local and state-level politics. Thus, these participants’ experiences should be understood within their particular geographic context. The present study indicates that additional research is needed to explore the barriers to and facilitators of LAI-ART in other social, political, and geographic contexts. Lastly, we selected participants for the qualitative sample based on their responses to the quantitative survey; we note the relatively wide window between the survey and their interview participation (35 to 387 days; M = 183.5 days). Consequently, some participants’ ART status, adherence, and overall level of care engagement were different by the time of the interviews. Importantly, though, the interviews still included a diversity of perspectives with regard to current adherence and viral suppression, as well as other aspects of lived experience. 

## 5. Conclusions

Our findings provide important and needed knowledge about the knowledge of, and willingness to use, LAI-ART among BSMM+, as well as the individual-, social-, and structural-level motivators of and barriers to LAI-ART. With equitable access and financial coverage, LAI-ART has the potential to overcome some known barriers to daily oral ART adherence. However, it is critical to explore the factors that influence whether new ART formulations reach those who can most benefit from them. Importantly, BSMM+ who are satisfied with their oral ART and have sustained viral suppression may not be an appropriate or necessary focus for new interventions seeking to increase LAI-ART uptake. Interventions should, instead, focus on those who want to make the switch for the convenience, or those who struggle with adherence and viral suppression, whether this be due to poor mental health, substance use, stigma, or because they simply forget to take doses from time to time. Understanding and addressing the barriers to LAI ART will improve the HIV clinical outcomes and increase our ability to achieve HIV health equity for diverse communities of BSMM+ in the US.

## Figures and Tables

**Table 1:** Participant demographics (*N* = 27).

				*n*	%
Age Mean	30.81		HIV Viral Load		
Median	31		Undetectable	21	78%
Range	26–35		Detectable	5	19%
			Don’t Know	1	4%
Time Since HIV Diagnosis (Years)					
Mean	10.85		Currently on ART?		
Median	10		Yes	23	85%
Range	6–17		No	4	15%
	*n*	%	On ART for Past 60 days	22	81%
Education					
High school diploma or GED	7	26%	Number of Days Missed ART Doses (Past 60 Days) **		
Some college, associate degree	14	52%	Never	6	27%
Bachelor’s degree	4	15%	Less than once a week	4	18%
Any graduate studies	2	7%	Once a week	2	9%
			2–3 days a week	6	27%
Sexual Identity			4–6 days a week	1	5%
Gay	21	78%	Every day	3	14%
Bisexual	6	22%			
Income			Missed ART ≥7 Consecutive Days (Past 12 months)? ^±^		
Less than USD 10,000	6	22%	Yes	6	26%
USD 10,000–19,999	4	15%	No	17	74%
USD 20,000–39,999	6	22%			
USD 40,000–59,999	6	22%	Ever Experienced a Relationship with IPV ^		
USD 60,000–79,999	2	7%	Yes	17	77%
Decline to Answer	1	4%	No	5	23%
Don’t know	2	7%			
			Substance Use (Past 60 Days)		
Insurance Status			Cocaine	6	22%
Yes	19	70%	Prescription Stimulants	7	26%
No	7	26%	Meth	9	33%
Unknown	1	4%	Inhalants	3	11%
			Sedatives/Sleeping Pills	4	15%
Employment			Hallucinogens	3	11%
Full Time	12	44%	Street Opioids	2	7%
Part-Time	4	15%	Prescription Opioids	2	7%
Unemployed	11	41%			

** Of those on ART for the past 60 days (*n* = 22); ^±^ Of those currently on ART (*n* = 23); ^ Of those who have ever been in a relationship (*N* = 22).

## Data Availability

The data by which these findings are supported can be requested from the corresponding author. The investigators will meet to review and respond to each request. The data are not publicly available due to concerns about participant privacy around some of the most sensitive domains of the interviews.

## References

[1] Centers for Disease Control and Prevention (2021). HIV Surveillance Report 2019.

[2] Centers for Disease Control and Prevention (2021). Monitoring Selected National HIV Prevention and Care Objectives by Using HIV Surveillance Data—United States and 6 Dependent Areas, 2019.

[3] Buchacz K., Armon C., Tedaldi E., Palella F.J., Novak R.M., Ward D., Hart R., Durham M.D., Brooks J.T., HIV Outpatient Study Investigators (2018). Disparities in HIV viral load suppression by race/ethnicity among men who have sex with men in the HIV outpatient study. AIDS Res. Hum. Retroviruses.

[4] Fedonni D., Buendia J.R., Sears S.C., Vaaler M.L., Mgbere O.O. (2021). Factors Associated with ART Adherence among MSM Receiving Medical Care in Texas: An Analysis of the Texas Medical Monitoring Project Data. J. Behav. Health.

[5] Shubber Z., Mills E.J., Nachega J.B., Vreeman R., Freitas M., Bock P., Nsanzimana S., Penazzato M., Appolo T., Doherty M. (2016). Patient-reported barriers to adherence to antiretroviral therapy: A systematic review and meta-analysis. PLoS Med..

[6] Tan J.Y., Campbell C.K., Conroy A.A., Tabrisky A.P., Kegeles S., Dworkin S.L. (2018). Couple-Level Dynamics and Multilevel Challenges Among Black Men Who Have Sex With Men: A Framework of Dyadic HIV Care. AIDS Patient Care STDS.

[7] Kerrigan D., Mantsios A., Gorgolas M., Montes M.-L., Pulido F., Brinson C., Devente J., Richmond G.J., Beckham S.W., Hammond P. (2018). Experiences with long acting injectable ART: A qualitative study among PLHIV participating in a Phase II study of cabotegravir+ rilpivirine (LATTE-2) in the United States and Spain. PLoS ONE.

[8] Boroughs M.S., Valentine S.E., Ironson G.H., Shipherd J.C., Safren S.A., Taylor S.W., Dale S.K., Baker J.S., Wilner J.G., O’cleirigh C. (2015). Complexity of childhood sexual abuse: Predictors of current post-traumatic stress disorder, mood disorders, substance use, and sexual risk behavior among adult men who have sex with men. Arch. Sex. Behav..

[9] Quinn K.G., Voisin D.R. (2020). ART adherence among men who have sex with men living with HIV: Key challenges and opportunities. Curr. HIV/AIDS Rep..

[10] Ramos S.D., Vincent W., Siconolfi D.E., Pollack L.M., Horvath K.J., Campbell C.K., Tebbetts S., Kegeles S.M., Storholm E.D. (2023). Differential associations of depressive symptomology to HIV care engagement among young black sexual minority men with HIV (YBSMM+) in the US South: A multi-group analysis of mood, intimate partner violence, and alcohol use. AIDS Behav..

[11] Quinn K.G., Spector A., Takahashi L., Voisin D.R. (2021). Conceptualizing the effects of continuous traumatic violence on HIV continuum of care outcomes for young Black men who have sex with men in the United States. AIDS Behav..

[12] Quinn K.G., Reed S.J., Dickson-Gomez J., Kelly J.A. (2018). An exploration of syndemic factors that influence engagement in HIV care among Black men. Qual. Health Res..

[13] Sweeney S.M., Vanable P.A. (2016). The association of HIV-related stigma to HIV medication adherence: A systematic review and synthesis of the literature. AIDS Behav..

[14] Rao D., Kekwaletswe T., Hosek S., Martinez J., Rodriguez F. (2007). Stigma and social barriers to medication adherence with urban youth living with HIV. AIDS Care.

[15] Rintamaki L., Kosenko K., Hogan T., Scott A.M., Dobmeier C., Tingue E., Peek D. (2019). The role of stigma management in HIV treatment adherence. Int. J. Environ. Res. Public Health.

[16] Kanazawa J.T., Saberi P., Sauceda J.A., Dubé K. (2020). The LAIs Are Coming! Implementation Science Considerations for Long-Acting Injectable Antiretroviral Therapy in the United States: A Scoping Review. AIDS Res. Hum. Retroviruses.

[17] Jolayemi O., Bogart L.M., Storholm E.D., Goodman-Meza D., Rosenberg-Carlson E., Cohen R., Kao U., Shoptaw S., Landovitz R.J. (2022). Perspectives on preparing for long-acting injectable treatment for HIV among consumer, clinical and nonclinical stakeholders: A qualitative study exploring the anticipated challenges and opportunities for implementation in Los Angeles County. PLoS ONE.

[18] Campbell C.K., Dube K., Sauceda J.A., Ndukwe S., Saberi P. (2022). Antiretroviral therapy experience, satisfaction, and preferences among a diverse sample of young adults living with HIV. AIDS Care.

[19] D’Amico R., Margolis D.A. (2020). Long-acting injectable therapy: An emerging paradigm for the treatment of HIV infection. Curr. Opin. HIV AIDS.

[20] Dubé K., Eskaf S., Evans D., Sauceda J., Saberi P., Brown B., Averitt D., Martel K., Meija M., Campbell D. (2020). The dose response: Perceptions of people living with HIV in the United States on alternatives to oral daily antiretroviral therapy. AIDS Res. Hum. Retroviruses.

[21] Balasubramanian R., Kasaie P., Schnure M., Dowdy D.W., Shah M., Fojo A.T. (2022). Projected impact of expanded long-acting injectable PrEP use among men who have sex with men on local HIV epidemics. JAIDS J. Acquir. Immune Defic. Syndr..

[22] Holloway I., Dougherty R., Gildner J., Beougher S.C., Pulsipher C., Montoya J.A., Plant A., Leibowitz A. (2017). PrEP uptake, adherence, and discontinuation among California YMSM using geosocial networking applications. J. Acquir. Immune Defic. Syndr..

[23] Tran N.K., Martinez O., Scheim A.I., Goldstein N.D., Welles S.L. (2022). Perceived Barriers to and Facilitators of Long-Acting Injectable HIV PrEP Use Among Black, Hispanic/Latino, and White Gay, Bisexual, and Other Men Who Have Sex with Men. AIDS Educ. Prev..

[24] Watson R.J., Morgan E., Sherman J., Caba A.E., Wheldon C.W., Chan P.A., Eaton L.A. (2023). Pre-exposure prophylaxis (PrEP) use, anticipated PrEP stigma, and bisexual identity among a Black and Hispanic/Latino sexual and gender diverse sample. Behav. Med..

[25] Wray T.B., Chan P.A., Klausner J.D., Ward L.M., Ocean E.M. (2022). Gay, bisexual, and other men who have sex with men who are not on oral PrEP may be less interested in available injectable products than in oral PrEP: Examining individual-level determinants of interest and barriers across products. AIDS Behav..

[26] Cooper S.E., Rosenblatt J., Gulick R.M. (2022). Barriers to uptake of long-acting antiretroviral products for treatment and prevention of Human Immunodeficiency Virus (HIV) in high-income countries. Clin. Infect. Dis..

[27] Coombs A., McFarland W., Ick T., Fuqua V., Buchbinder S.P., Fuchs J.D. (2014). Long-chain peer referral to recruit black MSM and black transgender women for an HIV vaccine efficacy trial. J. Acquir. Immune Defic. Syndr..

[28] Storholm E.D., Siconolfi D.E., Campbell C.K., Pollack L.M., Kegeles S.M., Rebchook G.M., Tebbetts S., Vincent W. (2023). Structural Inequities, Syndemics, and Resilience: The Critical Role of Social Support in Overcoming Barriers and Empowering Engagement in HIV Care for Young Black Sexual-Minority Men in the US South. J. Racial Ethn. Health Disparities.

[29] Vincent W., Siconolfi D.E., Pollack L., Campbell C.K., Kegeles S.M., Storholm E.D. (2023). What’s in Your Dataset? Measuring Engagement in HIV Care Using Routinely Administered Items with a Population Disproportionately Burdened by HIV. AIDS Behav..

[30] Malterud K., Siersma V.D., Guassora A.D. (2016). Sample size in qualitative interview studies: Guided by information power. Qual. Health Res..

[31] Braun V., Clarke V., Hayfield N., Davey L., Jenkinson E. (2023). Doing reflexive thematic analysis. Supporting Research in Counselling and Psychotherapy: Qualitative, Quantitative, and Mixed Methods Research.

[32] Braun V., Clarke V. (2022). Conceptual and design thinking for thematic analysis. Qual. Psychol..

[33] Braun V., Clarke V. (2006). Using thematic analysis in psychology. Qual. Res. Psychol..

[34] Braun V., Clarke V. (2023). Is thematic analysis used well in health psychology? A critical review of published research, with recommendations for quality practice and reporting. Health Psychol. Rev..

[35] King N., Brooks J. (2018). Thematic analysis in organisational research. The SAGE Handbook of Qualitative Business and Management Research Methods: Methods and Challenges.

[36] Goedel W.C., Nunn A.S., Chan P.A., Duncan D.T., Biello K.B., Safren S.A., Marshall B.D. (2019). A shot at equity? Addressing disparities among Black MSM in the coming era of long-acting injectable preexposure prophylaxis. AIDS.

[37] Levy M.E., Patrick R., Gamble J., Rawls A., Opoku J., Magnus M., Kharfen M., Greenberg A.E., Kuo I. (2017). Willingness of community-recruited men who have sex with men in Washington, DC to use long-acting injectable HIV pre-exposure prophylaxis. PLoS ONE.

[38] Patel R.R., Crane J.S., López J., Chan P.A., Liu A.Y., Tooba R., James A.S. (2018). Pre-exposure prophylaxis for HIV prevention preferences among young adult African American men who have sex with men. PLoS ONE.

[39] Simoni J.M., Beima-Sofie K., Mohamed Z.H., Christodoulou J., Tapia K., Graham S.M., Ho R., Collier A.C. (2019). Long-acting injectable antiretroviral treatment acceptability and preferences: A qualitative study among US providers, adults living with HIV, and parents of youth living with HIV. AIDS Patient Care STDS.

[40] Eaton L.A., Driffin D.D., Kegler C., Smith H., Conway-Washington C., White D., Cherry C. (2015). The role of stigma and medical mistrust in the routine health care engagement of black men who have sex with men. Am. J. Public Health.

[41] Meyers-Pantele S.A., Sullivan P., Mansergh G., Hirshfield S., Stephenson R., Horvath K.J. (2021). Race-based medical mistrust, HIV-related stigma, and ART adherence in a diverse sample of men who have sex with men with HIV. AIDS Behav..

[42] Orkin C., Arasteh K., Górgolas Hernández-Mora M., Pokrovsky V., Overton E.T., Girard P.-M., Oka S., Walmsley S., Bettacchi C., Brinson C. (2020). Long-acting cabotegravir and rilpivirine after oral induction for HIV-1 infection. N. Engl. J. Med..

[43] Overton E.T., Richmond G., Rizzardini G., Jaeger H., Orrell C., Nagimova F., Bredeek F., Deltoro M.G., Swindells S., Andrade-Villanueva J.F. (2020). Long-acting cabotegravir and rilpivirine dosed every 2 months in adults with HIV-1 infection (ATLAS-2M), 48-week results: A randomised, multicentre, open-label, phase 3b, non-inferiority study. Lancet.

[44] Swindells S., Andrade-Villanueva J.-F., Richmond G.J., Rizzardini G., Baumgarten A., Masiá M., Latiff G., Pokrovsky V., Bredeek F., Smith G. (2020). Long-acting cabotegravir and rilpivirine for maintenance of HIV-1 suppression. N. Engl. J. Med..

[45] Christopoulos K.A., Grochowski J., Mayorga-Munoz F., Hickey M.D., Imbert E., Szumowski J.D., Dilworth S., Oskarsson J., Shiels M., Havlir D. (2023). First demonstration project of long-acting injectable antiretroviral therapy for persons with and without detectable human immunodeficiency virus (HIV) viremia in an urban HIV clinic. Clin. Infect. Dis..

[46] Gandhi M., Hickey M., Imbert E., Grochowski J., Mayorga-Munoz F., Szumowski J.D., Oskarsson J., Shiels M., Sauceda J., Salazar J. (2023). Demonstration project of long-acting antiretroviral therapy in a diverse population of people with, H.I.V. Ann. Intern. Med..

[47] Texas Health and Human Services Texas HIV Medication Program (THMP) Medication Formulary and Maximum Quantities 2023. [Updated September 2023]. https://www.dshs.texas.gov/sites/default/files/hivstd/meds/files/Formulary2.pdf.

[48] Programs DoADA ADAP Directory: Texas 2023. https://adap.directory/texas#cab.

[49] Meyers K., Wu Y., Brill A., Sandfort T., Golub S.A. (2018). To switch or not to switch: Intentions to switch to injectable PrEP among gay and bisexual men with at least twelve months oral PrEP experience. PLoS ONE.

[50] Xavier Hall C.D., Smith J.C., Driggers R.A., Stoller B., Khan Z., Li J., Ignatius E., Siegler A.J. (2021). PrEParing for long-acting injectable PrEP in the South: Perspectives from healthcare providers in Georgia. AIDS Care.

